# The Overlooked Outcome Measure for Spinal Cord Injury: Use of Assistive Devices

**DOI:** 10.3389/fneur.2019.00272

**Published:** 2019-03-22

**Authors:** Giorgio Scivoletto, Giulia Galli, Monica Torre, Marco Molinari, Mariella Pazzaglia

**Affiliations:** ^1^Spinal Cord Unit, IRCCS Fondazione Santa Lucia, Rome, Italy; ^2^Spinal Rehabilitation Lab, IRCCS Fondazione Santa Lucia, Rome, Italy; ^3^Department of Psychology, La Sapienza University of Rome, Rome, Italy

**Keywords:** spinal cord injury, rehabilitative tools, survey, outcome measures, embodiment

## Abstract

Although several outcome measures are used to assess various areas of interest regarding spinal cord injuries (SCIs), little is known about the frequency of their use, and the ways in which they transform shared knowledge into implemented practices. Herein, 800 professionals from the International Spinal Cord Society, especially trained for caring in patients with SCI, were invited to respond to an Internet survey collecting information on the use of standardized measures in daily clinical practices. We asked both clinicians and researchers with different areas of interest about their use of functional outcome measures, and, in particular, which scales they habitually use to assess various aspects of clinical practice and rehabilitation. We selected a set of rating scales, which were validated for measuring SCIs (http://www.scireproject.com/outcome-measures). The results show that the areas of interest assessed by most of the participants were neurological status, upper limb, lower limb gait, pain, spasticity, self-care, and daily living. The most widely used rating scales were the spinal cord independence measure, the functional independence measure and the International Standards for Neurological Classification of Spinal Cord Injury. Instead, the majority of respondents did not evaluate the use of assistive technology. Despite the availability of several outcome scales, the practice of evaluating SCIs with standardized measures for assistive technologies and wheelchair mobility is still not widespread, even though it is a high priority in the rehabilitation of SCI patients. The results emphasize the need for a more thorough knowledge and use of outcome scales, thus improving the quality of assistive device evaluation.

## Introduction

Traumatic spinal cord injury (SCI) is a condition that affects nearly one out of every 1,000 people each year (0.721–0.906 per thousand people in the United States) and represents one of the leading causes of disability worldwide (1). Young adults are at the highest risk (2, 3). As all areas of the disfunctions of patients with SCI are capable of rehabilitation, therapies must be coordinated comprehensively and effectively to treat the medical, physiologic and psychological consequences of the injury. As such, SCI rehabilitation is complex and resource demanding with a costs that may vary from $53,000–88,000 USD (including the first admission and readmissions within the first two years after the lesion) or more, depending on the country and the severity of the lesion (4, 5). Given these high costs, it is necessary to monitor the efficacy and efficiency of rehabilitation with appropriate functional measure outcomes.

Furthermore, in the last two decades, research in the field of SCI included more than 900 clinical trials (https://clinicaltrials.gov). It is clear that the results of these trials need to be assessed with appropriate and validated instruments.

Accordingly, several studies (6, 7) recommended the routine collection of standardized outcome measures that represent a common language, allowing for the evaluation of the impact of rehabilitation, the outcomes of different populations and different intervention protocols. However, an agreement has yet to be made over which outcome measures should be used. It is evident that, due to the complexity of SCI rehabilitation, one single outcome measure is not sufficient to measure all the benefits of intervention, meaning a pool of instruments is needed. These outcome measures should be, if not specifically designed for SCI subjects, at least validated for this population, demonstrating good psychometric characteristics and ease of use and scoring. Moreover, although several outcome measures are used to assess various areas of interest to SCI patients, little is known about the frequency of their use.

Thus, this study had two main aims. First, we sought to evaluate how frequently validated assessment scales are used in SCI rehabilitation. We used the outcome measures suggested in the Spinal Cord Injury Rehabilitation Evidence (SCIRE) project available at http://www.scireproject.com/outcome-measures to assess and synthesize the frequency of use of outcome measures for rehabilitation interventions in SCI. SCIRE provides the measures of best practices applicable for reliability, sensitivity and validity to rehabilitation in individual with SCI, evaluating 1,600 published studies.

Second, we investigated the medical community's interest in the use and evaluation of alternative measures that report on best practices available for SCI rehabilitation. This raises an important question about how specialists measure outcomes and whether assessments should focus on outcomes that are relevant to the majority of patients as well as individual patients. Currently, studies have not been conducted on the instruments that experts in different centers have found to be the most useful as outcome indicators in the transitional care of SCI patients. Many centers select and combine several clinical outcome measures depending on their aims, but only some are related to SCIs. This reduces the efficiency of routine care, leads to inconsistent outcome measurements, and complicates the comparison of medical data.

A shared knowledge of the clinical outcome measures and implemented practices would not only improve interdisciplinary communication and support clinical education, it would also facilitate the planning and implementation of treatment for SCI patients.

## Methods

### Participants

All professionals in the ISCoS registry were sent an invitation to participate in the survey. Of the total 800 invitations sent, 70 were rejected (emails returned). A total of 110 surveys were returned on the first request and 33 after a reminder, giving a total of 143 and a response rate of 18%. Nine had complete demographic information but did not answer the questions and were therefore not considered for the present analyses. Within the overall sample of 134 respondents (49 ± 10.9 years), 51% of the participants were female (47 ± 8.5 years), 49% were male (51.5 ± 12.4 years), and they were comparable in age (*p* > 1). Participants came from all fields of rehabilitation expertise, with physicians representing the majority (81), as well as 29 physical therapists, 7 occupational therapists, 6 nurses, and 4 psychologists. For the remaining seven respondents, the occupation was unknown. The region returning the greatest number of surveys was Europe (65 respondents), 30 from America, 18 from Australia and New Zealand, 15 from Asia, and 7 from Africa. Almost two thirds (64%) of respondents were clinicians and researchers, 11% only researchers, and 25% only clinicians.

The study was approved by the ethics committee of Santa Lucia Foundation and was performed in accordance with the Declaration of Helsinki. Informed consent was obtained from participants.

### Survey Description

To select the outcome measures for these surveys, we used the Outcome Measures section of the SCIRE Project (http://www.scireproject.com/outcome-measures). This section presents 107 measures examined for psychometric properties (i.e., reliability, validity, and responsiveness) and recommended for use in treating SCI. We then prepared a survey using SurveyMonkey®, asking the participants which outcome measures they use, within different areas of interest, including assistive technology, community reintegration, lower limb gait, mental state, neurological status, other affected systems, quality of life, pain, sexual function, spasticity, secondary conditions, self-care, daily living, skin status, upper limb, and wheelchair mobility. For each area, we listed all the outcome measures identified by the SCIRE, allowing participants to indicate more than one measure if needed. We added two possible answers: “I do not assess this area” and “I do assess this area with other measures.” When choosing the last answer, a box opened where the respondent could specify which measure they used for that area. We also asked for some demographic information, including the age, gender, geographical area, and type of work of the respondent as well as the center in which the respondent worked.

### Analysis

The survey responses were analyzed using descriptive statistics, such as frequencies and proportions. To better characterize the rehabilitation outcome measures identified by SCIRE, the distribution of different responses was subjected to hierarchical cluster analysis in which the responses were sorted according to their adhesion to the following responses: (i) “I do not assess this area,” (ii) “I assess with measures identified by SCIRE,” and (iii) “I assess with other measures.” Using cluster analysis, similar responses were grouped into homogeneous subsets. In this case, the analysis identified the measures with minimal within-response variation and maximal between-response variation. Hierarchical cluster analysis does not require pre-specify selection of the number of clusters, and the dendrogram provides a simple and comprehensive image of the number of clusters. The analysis of the contingency table using the chi square test was employed to compare the frequency of each response.

## Results

### Cluster Analysis

**Figure 1:**
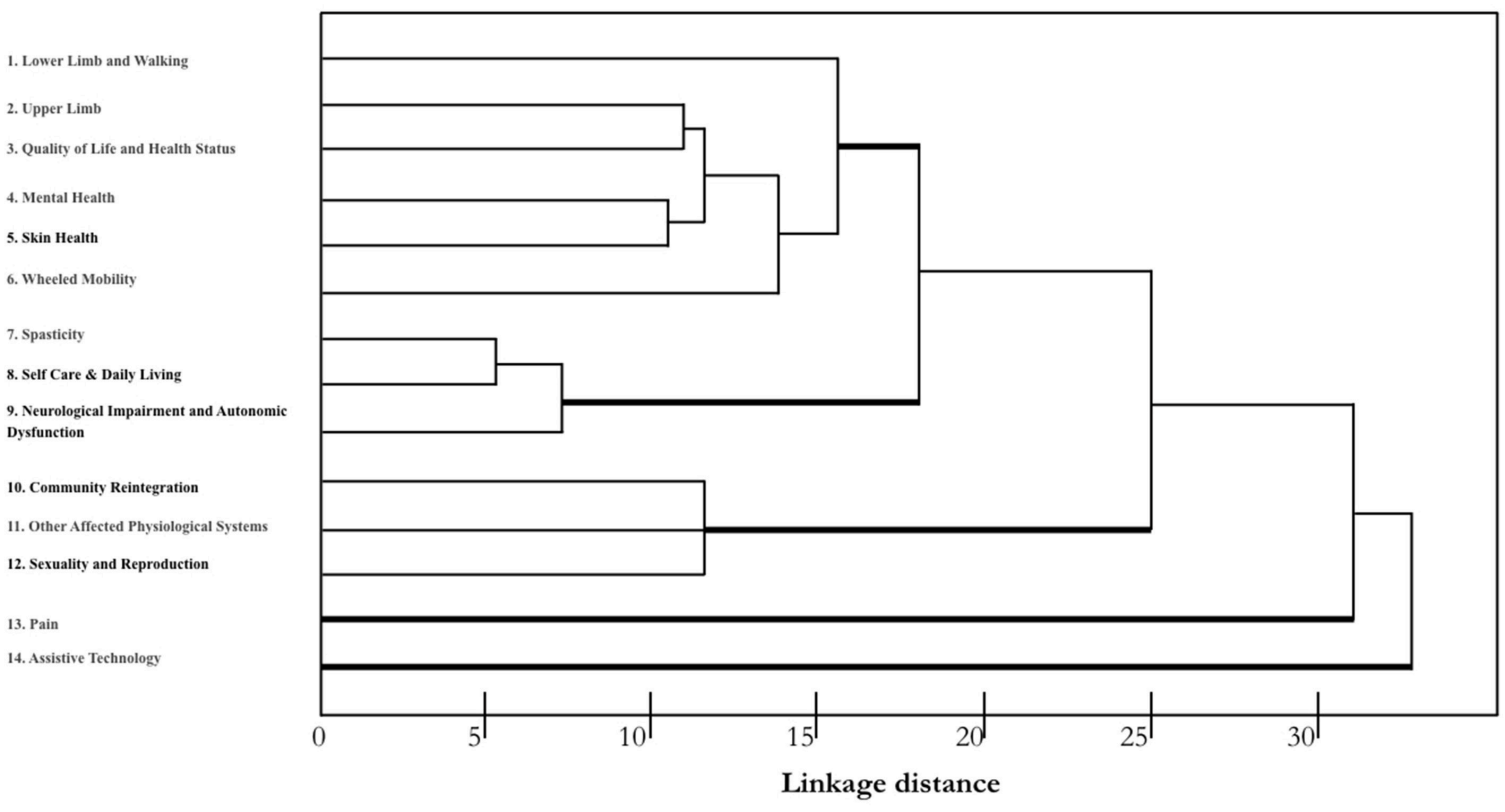
Dendrogram indicating the greatest difference between the responses of using those outcome measures identified by SCIRE. A single-linkage hierarchical clustering algorithm was used. The *x*-axis shows Euclidean distances that provide a measure of similarity in the distribution of responses. The types of measures are reported along the *y*-axis. Measures with the most similar performance are closer to each other. Three main clusters are apparent with relatively homogeneous measures. Pain and Assistive Technology clustered separately and seem to have a different profile.

A hierarchical cluster analysis of different responses was conducted to identify the varying degrees of (dis)similarity in the responses using a (dis)similarity matrix. The distribution of the different measures is shown in Figure 1.

**Figure 2:**
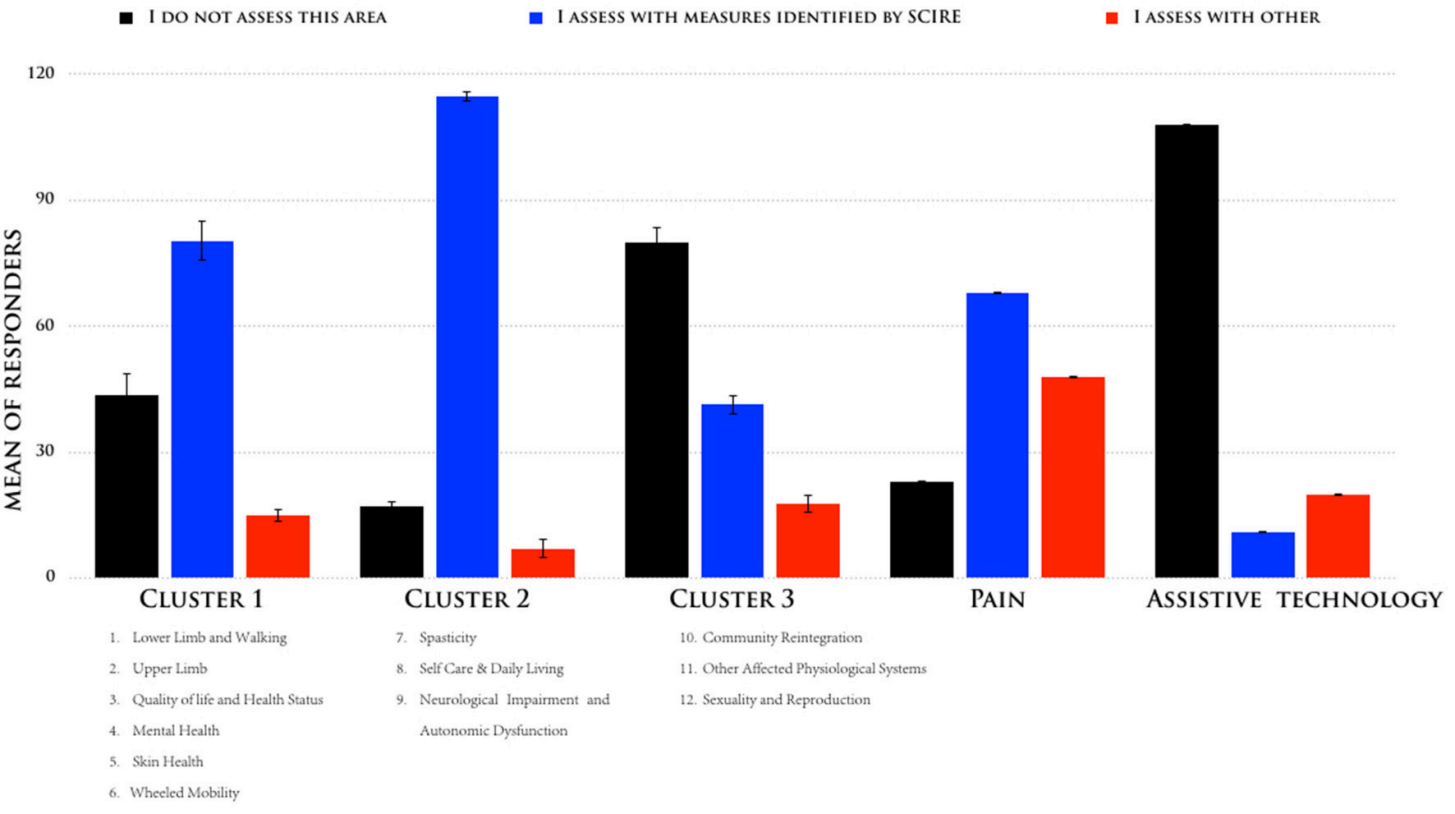
Means of the responses were sorted according to their adhesion to the following responses: (i) “I do not assess this area,” (ii) “I assess with measures identified by SCIRE,” and (iii) “I assess with other measures for the five different clusters”.

According to the described sorting process, we identified three clusters that had the greatest difference between the responses, and we compared them to the outcome measures identified by SCIRE: one cluster with three measures (nos. 7–9) had a high consensus (mean = superior of 80%), another cluster with six measures (nos. 1–6) had a moderate consensus (mean = 58%), and one cluster with three measures had a low consensus (nos. 10–12). Figure 2 shows the means of the participants' responses for the three different clusters. While Pain and Assistive Technology were present, these were clustered as separate profiles. Pain presented as being separate from the other clusters for a moderate consensus (mean = 46%) of the respondents in their use of the SCIRE measures, but also for a moderate consensus (mean = 35%) in the use of alternative measures. This pattern is not present in the other clusters. Remarkably, Assistive Technology clustered as a separate profile mainly because the respondents did not assess this measure (mean = 85%). The analysis of the contingency table using the chi-square test indicated a significant difference among the three responses in the five clusters that were identified (χ^2^ = 175, *p* < 0.0001).

### Objective Outcome Measures Used in Patients With SCI

We determined the frequency of use of each measure indicated in set of outcome measurement of SCIRE Toolkit. The most commonly used objective outcome measures were: the International Standards for Neuorlogical Classification of Spinal Cord Injury designed to assess the Neurological Impairment, and the MAS developed to evaluate Spasticity in more of 80% of the responders, the SCIM and FIM in clinical area of Self Care and Daily Living and 6MWT, 10 MWT, WISCI for clinical area lower limb and walking in more of 50% of participants. Table 1 presents a list of the measures that the respondents most commonly selected as their first and second choice for each clinical area using the SCIRE toolkit.

**Table 1:** Rank order of preferred outcome measure by clinical area of use.

**Clinical area**	**Outcome measures selected**		**Set of outcome measurement of SCIRE toolkit**
	**1° choice**	**2° choice**	
Assistive technology	Quebec user evaluation of satisfaction with assistive technology	Assistive technology device predisposition assessment	• Assistive technology device predisposition assessment • Quebec user evaluation of satisfaction with assistive technology
Community reintegration	Craig handicap assessment and reporting technique	Community integration questionnaire	• Assessment of Life Habits Scale (LIFE-H) • Community Integration Questionnaire (CIQ) • Craig Handicap Assessment and Reporting Technique (CHART) • Impact on Participation and Autonomy Questionnaire (IPAQ) • Physical Activity Recall Assessment for People with Spinal Cord Injury (PARA-SCI) • Reintegration to Normal Living (RNL) Index • Physical Activity Scale for Individuals with Physical Disability (PASIPD)
Lower limb and walking	6-Min walk test	10 Meter walking test	• 6-Min Walk Test (6MWT) • 10 Meter Walking Test (10 MWT) • Berg Balance Scale (BBS) • Clinical Outcome Variables Scale (COVS) • Functional Standing Test (FST) • Spinal Cord Injury Functional Ambulation Inventory (SCI-FAI) • Timed Up and Go Test (TUG) • Walking Index for Spinal Cord Injury (WISCI) and WISCI II
Mental health	Hospital anxiety and depression scale	Beck depression inventory	• Beck Depression Inventory (BDI) • Brief Symptom Inventory (BSI) • CAGE Questionnaire • Center for Epidemiological Studies Depression Scale (CES-D and CES-D-10) • Depression Anxiety Stress Scale-21 (DASS-21) • Fatigue Severity Scale (FSS) • Scaled General Health Questionnaire-28 (GHQ-28) • Hospital Anxiety and Depression Scale (HADS) • Patient Health Questionnaire-9 (PHQ-9) • Symptom Checklist-90-Revised (SCL-90-R) • Zung Self-Rating Depression Scale (SDS)
Neurological impairment and autonomic dysfunction	International standards for neuorlogical classification of spinal cord injury	Surface electromyography	• International standards for neurological classification of spinal cord injury • Surface electromyography
Other affected physiological systems	Spinal cord injury secondary conditions scale	Spinal cord lesion coping strategies questionnaire	• Spinal Cord Injury Secondary Conditions Scale (SCI-SCS) • Exercise Self-Efficacy Scale (ESES) • Moorong Self-Efficacy Scale (MSES) • Spinal Cord Lesion Coping Strategies Questionnaire (SCL CSQ) • Spinal Cord Lesion Emotional Well-being Questionnaire (SCL EWQ) • Wingate Anaerobic Testing (WAnT)
Pain	Classification system for chronic pain in SCI	Brief pain inventory	• Classification system for chronic pain in SCI • Donovan SCI Pain Classification System • Multidimensional Pain Inventory (MPI) - SCI version • Multidimensional Pain Readiness to Change Questionnaire (MPRCQ2) • Quantitative Sensory Testing (QST) • Tunk's Classification Scheme • Wheelchair Users Shoulder Pain Index (WUSPI) • Brief Pain Inventory (BPI)
Quality of life and health status	Short form 36	Life satisfaction questionnaire	• Incontinence Quality of Life Questionnaire (I-QOL) • Life Satisfaction Questionnaire (LISAT-9, LISAT-11) • Quality of Life Index (QLI) - SCI Version • Quality of Life Profile for Adults with Physical Disabilities (QOLP-PD) • Quality of Well-Being (QWB) and Quality of Well-Being—Self-Administered (QWB-SA) • Qualiveen • Satisfaction with Life Scale (SWLS, Deiner Scale) • Short Form 36 (SF-36) • Sickness Impact Profile 68 (SIP 68) • World Health Organization Quality of Life—BREF (WHOQOL-BREF)
Self care and daily living	Spinal cord independence measure	Functional independence measure	• Appraisal of DisAbility: Primary and Secondary Scale (ADAPSS) • Rivermead Mobility Index (RMI) • Barthel Index (BI) • Frenchai Activities Index (FAI) • Functional Independence Measure (FIM) • Functional Independence Measure Self-Report (FIM-SR) • Klein-Bell Activities of daily Living scale (K-B Scale) • Lawton Instrumental Activities of Daily Living scale (IADL) • Quadriplegia Index of Function—Short Form (QIF-SF) • Self Care Assessment Tool (SCAT) • Self Reported Functional Measure (SRFM) • Spinal Cord Injury Lifestyle Scale (SCILS) • Spinal Cord Independence Measure (SCIM) • Quadriplegia Index of Function Modified (QIF-Modified)
Sexuality and reproduction	Sexual interest and satisfaction scale	Sexual behavior scale (SBS)	• Emotional Quality of the Relationship Scale (EQR) • Knowledge, Comfort, Approach, and Attitude toward Sexuality Scale (KCAASS) • Sexual Attitude and Information Questionnaire (SAIQ) • Sexual Behavior Scale (SBS) • Sexual Interest, Activity and Satisfaction (SIAS)/Sexual Activity and Satisfaction (SAS) Scales • Sexual Interest and Satisfaction Scale (SIS)
Skin health	Braden scale	Spinal cord injury pressure ulcer scale measure	• Skin Management Needs Assessment Checklist (SMNAC) • Abruzzese scale • Braden scale • Gosnell measure • Norton measure • Spinal Cord Injury Pressure Ulcer Scale (SCIPUS) Measure • Spinal Cord Injury Pressure Ulcer Scale—Acure (SCIPUS-A) • Stirling's pressure ulcer severity scale • Waterlow scale
Spasticity	Ashworth and modified ashworth scale	Penn spasm frequency scale (PSFS)	• Pendulum Test (Wartenberg) • Ashworth and Modified Ashworth Scale (MAS) • Penn Spasm Frequency Scale (PSFS) • Spinal Cord Assessment Tool for Spastic Reflexes (SCATS) • Spinal Cord Injury Spasticity Evaluation Tool (SCI-SET)
Upper limb	Hand-held myometer	Grasp and release test	• Box and Block Test (BBT) • Capabilities of Upper Extremity Instrument (CUE) • Grasp and Release Test (GRT) • Hand-Held Myometer • Jebsen Hand Function Test (JHFT) • Modified Functional Reach Test (mFRT) • Sollerman hand function test • Tetraplegia Hand Activity Questionnaire (THAQ) • Van Lieshout Test Short Version (VLT-SV) • Graded Redefined Assessment of Strength, Sensibility, and Prehension (GRASSP) • 6-Min Arm Test (6-MAT)
Wheeled mobility	Wheelchair skills tests	Wheelchair circuit	• 4 Functional Tests for Persons who Self-Propel a Manual Wheelchair (4FTPSMW) • Tool for assessing mobility in wheelchair-dependent paraplegics • Timed Motor Test (TMT) • Wheelchair Circuit (WC) • Wheelchair Skills Tests (WST) • I don't assess Wheeled Mobility

### The Use of Measures Outcome in Europe and America

We also assessed the differences in outcome measures identified by SCIRE when considering regions with universal healthcare (Canada and Europe) vs. fee-for-service healthcare (USA). An analysis of the contingency table using chi square test indicated a significant difference among the three responses in four measures identified:

For Community (χ^2^ = 4.21, *p* < 0.04), Self-care (χ^2^ = 3.75, *p* = 0.05), and Quality of life (χ^2^ = 4.48, *p* < 0.03), the difference is explained given that compared to USA, Europe has less centers that assess the outcomes in these measures.

For Pain, meanwhile, American centers had a high consensus of using the SCIRE outcome measures. The data also showed that Europe centers had a substantial proportion of the respondents answer that they use “other measures.” In other measures, respondents indicated the Visual Analog Scale or the Numeric Rating Scale, quick, and easy measures to use to assess the pain.

No differences in Assistive Technology were present between the regions with and without fee-for-service healthcare because the outcome measure was not evaluated in rehabilitation centers.

## Discussion

The data suggested that, for motor and sensory impairment, the consensus among professionals is to assess SCI by using the outcome measures identified by the SCIRE. Although better standardized outcome measures have been selected by the SCIRE in the treatment of SCI patients, a number of the identified areas are under-evaluated with regards to some recommended measures, while other areas are not evaluated at all in rehabilitation centers. The survey's most striking result is that the use of assistive devices is not currently studied by rehabilitation specialists as an outcome measure.

### Less Widely Used Rating Scales for Spinal Cord Injury

The majority of respondents did not evaluate the use of Assistive Technology as an outcome measure identified by the SCIRE. We investigated whether the respondents chose another measure specifically to do with the use of assistive devices, different from those selected by the SCIRE. Interestingly, no other measures were used to study the use of assistive devices, suggesting that this outcome is of little interest to medical professionals and researchers. The reasons for this could include this outcome measure's failure to adequately reflect recovery after SCI, the lack of valid instruments to measure this outcome, the requirement to have a primary outcome area to support self-care in SCI treatment, and the short time period allotted to study this outcome measure and to care for patients. Unlike other outcome measures, the decision to use a functional measure for the use of assistive devices seems mainly to be at the discretion of the treating medical professional or researcher, instead of rehabilitation specialists. This could represent a bias for several reasons.

First, providing mobility equipment that meets the individual needs of an SCI patient encourages that patient to be independent and to participate in society while reducing that patient's behavioral challenges and reliance on assistance (8, 9). Conversely, inappropriate mobility equipment can restrict an individual's independence and opportunities for a social life (10–12). Therefore, assigning assistive devices to SCI patients is challenging. Selecting an appropriate prosthetic device, training for its initial use, and evaluating for a possibly more suitable devices and overall outcomes are essential aspects of rehabilitation.

Furthermore, the strong connection between tool and body perception, often termed embodiment (8, 9, 11, 13–17), may be one of the most crucial factors affecting functional recovery (18–22). Recent studies have demonstrated that establishing a sense of embodiment for prostheses in patients with limb amputation is associated with enhanced competence and patients' ease of use of such devices. Conversely, a low level of embodiment impedes the efficient use of assistive tools and contributes to their rejection (11, 23). Therefore, surveys to measure technology acceptance should always be conducted on SCI patients (17).

Third, in recent years, the focus on assistive devices that use robotic technologies to aid in recovery and rehabilitative treatment has increased (24–26). Adequate provisions, for example walking with exoskeleton, can reduce clinical complications resulting from life in a wheelchair, decrease the intensity of pain and spasticity, increase bone density, and improve well-ness and the overall quality of life (27). While substantial advancements have been made in terms of the portability and safety of assistive devices, little attention has been devoted to the outcome measures that must be studied for their usage. Despite great progress from a technological standpoint, as well as SCI patients facing medical and societal pressures to move in wheelchairs or use other assistive tools, only a small number of centers assess outcome measures related to assistive devices.

Besides Assistive Technology, the results of the present survey show that a number of areas identified by the SCIRE are evaluated less than others (Other Affected Systems, Sexuality, and Reproduction). It is still possible that being physicians the majority of respondents, they might not have the same comprehensive knowledge for all the areas surveyed.

Possible other reasons for not collecting these outcome measures may include: resource constraints and the lack of a consensus among professionals regarding which outcome measures should be used (28). Furthermore, some instruments are never or are seldom used due to inadequate measures. A different possible explanation is that the extent of the use of one measure could be considered an indicator of its usefulness (29). Although this assumption has yet to be proven, the little use of some measures might suggest that clinicians do not rely on these measures to assess the outcome of an intervention (30).

### Most Widely Used Outcome Measures for Spinal Cord Injury

At present, no comprehensive survey has been conducted within the field of SCI other than the one conducted in the current study. The only possible comparisons are a Canadian survey on amputees (30) and a United Kingdom (28) survey on rehabilitation centers. According to these two surveys, the self-care measure is the most frequently assessed (75–80% of the respondents in the previous studies, 87% in the present one). The functional independence measure (FIM) and the spinal cord independence measure (SCIM) were the most frequently used measures for self-care and daily living, with the SCIM being the most popular at 58% and the FIM close behind at 50%. This likely reflects the different origins of the respondents, with most respondents being European. This could also be due to the increasing success of the SCIM in the field of SCI. In fact, not only did the SCIM prove to be more sensitive to the changes of SCI patients than the FIM (31), it is also recognized as the first choice outcome measure (32, 33). Although the FIM requires significant time for training and data collection, it remains a widely used measure in some countries. However, it is possible that this popularity is partly because the FIM must be used in some countries due to administrative requirements.

The International Standards for Neurological Classification of Spinal Cord Injury (ISNCSCI) published by the American Spinal Injury Association is a well-established international outcome measure utilized by both researchers and clinicians to quantify the level of neurological impairment resulting from an SCI. One of the common complications for SCIs is spasticity, which has received some attention with the Ashworth and Modified Ashworth scales (MAS). The MAS appears to provide a valid scale for qualitative assessments that can easily be used in practice.

Other areas that are frequently assessed include lower limb and walking, which was evaluated by more than 78% of the respondents. In this area, the measures used most were two time tests (i.e., the 10-meter walk test and the 6-min walk test) and the Walking Index for Spinal Cord Injury (WISCI). This is in agreement with what was suggested by the SCIRE and several guidelines (34, 35). The WISCI assesses walking capacity based on the need for orthosis, walking aids, and assistance (36). However, it does not offer any information on walking speed and suffers from a ceiling effect (i.e., those who are level 20 cannot improve further) that is reached by SCI subjects within the first 6 months after the lesion (37). The timed tests describe walking in terms of speed and endurance but suffer from a floor effect, as patients who are unable to walk cannot be assessed. Therefore, the combination of these three measures (WISCI, MAS, ASIA) seems to be the best method to assess the walking and walking improvements of SCI patients.

The Upper Limb area is frequently assessed as well, but using a variety of measures that makes it difficult to suggest a preferred measure. However, it should be noted that half of the respondents who answered “Other” declared that they assessed the upper limb area using a strength assessment. Together with the 18% of respondents who used a handheld myometer (38), in total, 30% of respondents assessed the upper limb area using a strength assessment. This is probably because most of the proposed tests require a number of staff or resources as well as time, training, and special equipment that is not always free available.

### Outcome Measures for Pain

One peculiar result of this survey concerns the use of outcome measures for pain. In total, 40% of the respondents answered that they used measures other than those proposed. Most of them used the visual analog scale [VAS (39)] or the numeric rating scale [NRS (40)] instead of or in addition to other rapid measures to assess pain. American centers showed a high consensus in using the SCIRE. At present, no measure (at least within those listed by SCIRE) takes all aspects of pain into account. Some examine the nature and localization of the pain, some examine the impact of the pain on a patient's life, and some examine only particular aspects of pain [e.g., the Wheelchair User's Shoulder Pain Index (WUSPI)]. Furthermore, most of these tests are time consuming. Although the International Spinal Cord Injury Pain Classification indicates several distinct types of pain, including neuropathic, nociceptive, other, and unknown pain (41), only 25% of the respondents indicated using the Classification System for Chronic Pain in SCI and Donovan SCI Pain Classification System proposed by SCIRE to distinguish between neuropathic and nociceptive pain. Most of respondents answered that they used DN4 than those proposed. Most of respondents answered that they used DN4 instead of the measures that were proposed.

However, the VAS and the NRS can be conducted quickly and thus could be utilized for repeated assessments during the day to gauge the severity of the pain and the effects of drugs and treatments.

It should be noted that the SCIRE does not list between the outcome measures for pain in the International Spinal Cord Injury Pain Basic Data Set (42), probably because it is still quite new and is not widespread or commonly utilized in research settings. This instrument, produced by the ISCoS, has the advantage of encompassing different aspects of pain (i.e., type, localization, intensity, and impact on daily life activities and mood), thus reconciling the flaws of other instruments listed by the SCIRE. This type of evaluation may be very useful in patients with SCI who may present with pain due to a primary neurological involvement or to other non-neurological causes, or even mixed pain (for example pain associated with spasticity or painful tonic spasms). To define the exact type of pain it is necessary to target a more specific pharmacological treatment.

### Limitations

This study has some limitations. Of the 800 SCI clinicians and researchers who were invited several times to participate in the survey, only 143 completed it. Although they represented clinicians from most countries around the world (most of the respondents were from Europe), the low number of participants could limit the generalizability of our results. However, this response rate is typical of surveys that examine clinical practice (43–45). The second limitation is the different experiences of respondents (mostly physicians, but also physical therapists, occupational therapists, nurses, and psychologists). It is also difficult to say whether the respondents had comprehensive knowledge of all the areas surveyed. Future studies addressing multiple confounding factors are necessary to establish which factors improve the outcomes of SCI patients. However, this study suggests the need to generate new knowledge regarding the outcome measures of assistive devices as well as the impact of adopting a new approach when using assistive devices in cases of brain–body disconnection, opening the door to an innovative clinical prospect in terms of user-centered neuroprosthetic technologies (46).

## Data Availability

The datasets generated for this study are available on request to the corresponding author.

## Author Contributions

GS and MP conceived and designed research, interpreted the results, drafted the manuscript. GS, GG, and MP edited and revised the manuscript. MT and MM critical revision of manuscript for intellectual content. All authors have approved the final version of the manuscript.

### Conflict of Interest Statement

The authors declare that the research was conducted in the absence of any commercial or financial relationships that could be construed as a potential conflict of interest. The reviewer MP declared a shared affiliation, with no collaboration, with one of the authors, MP, to the handling editor at time of review.
